# Evidence for the evolutionary steps leading to *mecA*-mediated β-lactam resistance in staphylococci

**DOI:** 10.1371/journal.pgen.1006674

**Published:** 2017-04-10

**Authors:** Joana Rolo, Peder Worning, Jesper Boye Nielsen, Rita Sobral, Rory Bowden, Ons Bouchami, Peter Damborg, Luca Guardabassi, Vincent Perreten, Henrik Westh, Alexander Tomasz, Hermínia de Lencastre, Maria Miragaia

**Affiliations:** 1 Laboratory of Molecular Genetics, Instituto de Tecnologia Química e Biológica António Xavier, Universidade Nova de Lisboa (ITQB-NOVA), Oeiras, Portugal; 2 Laboratory of Bacterial Evolution and Molecular Epidemiology, Instituto de Tecnologia Química e Biológica António Xavier, Universidade Nova de Lisboa (ITQB-NOVA), Oeiras, Portugal; 3 MRSA Knowledge Center, Department of Clinical Microbiology, Hvidovre Hospital, Hvidovre, Denmark; 4 UCIBIO-REQUIMTE, Departamento de Ciências da Vida, Faculdade de Ciências e Tecnologia, Universidade Nova de Lisboa, Caparica, Portugal; 5 Department of Statistics, University of Oxford, Oxford, United Kingdom; 6 Department of Veterinary Disease Biology, Faculty of Health and Medical Sciences, University of Copenhagen, Copenhagen, Denmark; 7 Department of Biomedical Sciences, Ross University School of Veterinary Medicine, Basseterre, St Kitts, West Indies; 8 Department of Molecular Epidemiology and Infectious Diseases, Institute of Veterinary Bacteriology, Vetsuisse Faculty, University of Bern, Bern, Switzerland; 9 Department of Clinical Medicine, Faculty of Health and Medical Sciences, University of Copenhagen, Copenhagen, Denmark; 10 Laboratory of Microbiology and Infectious Diseases, The Rockefeller University, New York, United States of America; Uppsala University, SWEDEN

## Abstract

The epidemiologically most important mechanism of antibiotic resistance in *Staphylococcus aureus* is associated with *mecA–*an acquired gene encoding an extra penicillin-binding protein (PBP2a) with low affinity to virtually all β-lactams. The introduction of *mecA* into the *S*. *aureus* chromosome has led to the emergence of methicillin-resistant *S*. *aureus* (MRSA) pandemics, responsible for high rates of mortality worldwide. Nonetheless, little is known regarding the origin and evolution of *mecA*. Different *mecA* homologues have been identified in species belonging to the *Staphylococcus sciuri* group representing the most primitive staphylococci. In this study we aimed to identify evolutionary steps linking these *mecA* precursors to the β-lactam resistance gene *mecA* and the resistance phenotype. We sequenced genomes of 106 *S*. *sciuri*, *S*. *vitulinus* and *S*. *fleurettii* strains and determined their oxacillin susceptibility profiles. Single-nucleotide polymorphism (SNP) analysis of the core genome was performed to assess the genetic relatedness of the isolates. Phylogenetic analysis of the *mecA* gene homologues and promoters was achieved through nucleotide/amino acid sequence alignments and mutation rates were estimated using a Bayesian analysis. Furthermore, the predicted structure of *mecA* homologue-encoded PBPs of oxacillin-susceptible and -resistant strains were compared. We showed for the first time that oxacillin resistance in the *S*. *sciuri* group has emerged multiple times and by a variety of different mechanisms. Development of resistance occurred through several steps including structural diversification of the non-binding domain of native PBPs; changes in the promoters of *mecA* homologues; acquisition of SCC*mec* and adaptation of the bacterial genetic background. Moreover, our results suggest that it was exposure to β-lactams in human-created environments that has driven evolution of native PBPs towards a resistance determinant. The evolution of β-lactam resistance in staphylococci highlights the numerous resources available to bacteria to adapt to the selective pressure of antibiotics.

## Author summary

The emergence and rise of *mecA*-mediated β-lactam resistance in staphylococci has been one of the greatest concerns of the scientific and medical communities worldwide. However, little is known regarding the origin of the *mecA* gene determinant. In this study we demonstrate that antibiotic pressure in the human environment and in food additives used in livestock was the major driving force of the evolution and spread of resistance to β-lactams. Furthermore, we confirm the previous findings suggesting that the development of resistance occurs in primitive species of staphylococci through diversification of a native penicillin binding protein involved in cell wall synthesis. We also demonstrate that resistance was achieved through four distinct mechanisms: accumulation of substitutions in a specific domain of the protein; diversification of the promoter of the gene; acquisition of SCC*mec*, and adaptation of the genetic background. Our results highlight the resources that primitive bacteria used to thrive in a changing environment that has led to the methicillin-resistant *Staphylococcus aureus* (MRSA) pandemics.

## Introduction

The most important antibiotic resistance mechanism in staphylococci is associated with the *mecA* gene, which confers resistance to the large class of β-lactam antibiotics. *mecA* is carried on a mobile genetic element called staphylococcal cassette chromosome *mec* (SCC*mec*) [1], which always inserts at the same locus in the chromosome, in the 3’ end of *orfX* (which encodes a RNA methyltransferase) [1, 2]. Several studies have demonstrated that acquisition of *mecA* confers to staphylococci a competitive advantage in the hospital, community and veterinary environments [3, 4]. Introduction of the *mecA* determinant into the *S*. *aureus* genome on multiple occasions, has led to the emergence and worldwide dissemination of several methicillin-resistant *S*. *aureus* (MRSA) clones [5].

The *mecA* determinant encodes an extra penicillin-binding protein (PBP2a). The expression of resistance is achieved by a slow rate of acylation of PBP2a as well as a low affinity of the enzyme for β-lactams [6]. Structural studies have revealed that the poor acylation rate, that PBP2a presents when in contact with β-lactams, is due to a distorted active site, provided by the flexibility of the non-binding (NB) domain and regions surrounding the active site groove in the transpeptidase (TP) domain [7]. Furthermore, the position of Ser403 is crucial for the nucleophilic attack of the β-lactam ring, which leads to acylation of the protein [7].

The first clinical MRSA isolates were identified in the UK in 1961, shortly after the introduction of methicillin into clinical practice [8, 9]. Early MRSA were found to present a heterogeneous profile of resistance to β-lactams [10]. Further studies have revealed that mutations in genes associated with cell division as well as central metabolism (the so-called auxiliary genes) influence the expression of β-lactam resistance and the resulting phenotype [11]. Moreover, the expression of homogeneous high level resistance has been associated with the activation of the bacterial stringent response, provoked by mutations in the *relA* system [12, 13] and related regulons and genes [14]. These findings underline the importance of the *S*. *aureus* genetic background in the expression of β-lactam resistance.

The rapid emergence of MRSA raised the hypothesis that *mecA* was already present in the staphylococcal gene pool prior to the introduction of methicillin. In fact, a ubiquitous homologue named *mecA1*, with 80% nucleotide identity to *mecA* has been identified in the primitive coagulase-negative *Staphylococcus sciuri* [15]. Several lines of evidence suggest that *mecA1* is the precursor of *mecA*. While *mecA1* does not confer resistance to β-lactams in *S*. *sciuri*, there are reports of β-lactam-resistant strains that have alterations in the promoter region of this gene [16]. When introduced experimentally into a *S*. *aureus* strain, *mecA1* was able to confer β-lactam resistance and produce a protein with properties resembling that of MRSA PBP2a [17, 18]. Additional *mecA* homologues have been identified in related species, including a *mecA* homologue (*mecA2*) with 90% nucleotide identity with *mecA* in *Staphylococcus vitulinus* [19]. Furthermore, *mecA* along with its regulators, *mecI* and *mecRI*, has been identified in a small number of *Staphylococcus fleurettii* isolates [20].

Despite the importance of *mecA* in the epidemiology of antibiotic resistant staphylococci, the evolutionary history of this gene has remained unclear. The purpose of this study was to shed light on the evolutionary steps linking the native *mecA* homologues identified in primitive coagulase negative staphylococci to the β-lactam resistance gene *mecA* and the resistance phenotype.

## Results

### Homologues of *mecA* are abundant in *S*. *sciuri*, *S*. *vitulinus* and *S*. *fleurettii*

The putative precursor of *mecA* is *mecA1*, previously shown to be ubiquitous in *S*. *sciuri* (15), but the frequency of the other *mecA* homologues (*mecA2* and *mecA*) in the remaining species of the *S*. *sciuri* group remained unclear. Additionally, the location of *mecA* homologues in the chromosome was unknown.

A search for *mecA* homologues by BLAST analysis in the genomes of 106 *S*. *sciuri*, *S*. *vitulinus* and *S*. *fleurettii* isolates collected from humans and animals showed that all strains carried at least one copy of *mecA* homologue. These were found either in the *orfX* (SCC*mec* insertion site) or 200 kb from *orfX*, a site that from now on, we will call *native location*. We confirmed that, in our collection, *mecA1* was present in all *S*. *sciuri* isolates [15] and *mecA* was present in all *S*. *fleurettii* strains [20] at the native location. *S*. *vitulinus* was different from the other species, since half of the strains (n = 9) carried *mecA2* [19], and the remaining strains either carried *mecA* (n = 6) or did not carry any *mecA* homologue in this region (n = 3).

### *mecA1* is a hot spot for diversification

Alignment of all *mecA1*, *mecA2* and *mecA* sequences (S1A Fig) showed that *mecA1* was extremely diverse, including a total of 44 different alleles (SID = 97.2%, CI = 95.7%-98.7%) that varied between 93–100% in nucleotide identity (S1B Fig). In contrast, *mecA2* and *mecA* were highly conserved (*mecA2*: SID = 70.4%, CI = 60.5%-80.2%; *mecA*: SID = 21.6%, CI = 9.7%-33.6%) varying from 99.75 to 100% in nucleotide identity.

Furthermore, amino acid sequence predictions showed that the SID of *mecA1-*encoded PBP4 was still very high, 96.2% (CI = 94.5%-97.9%). Interestingly, although both the nonbinding (NB) and transpeptidase (TP) domains were under purifying selection (dN/dS<1), the NB domain accumulated many more amino acid substitutions (36%) and showed a higher dN/dS per site (0.19) than the TP domain (8%; dN/dS = 0.05).

The genetic diversity observed for *mecA1* appears to have resulted both from recombination and mutation events, wherein the average recombination/mutation rate/site was estimated to be 0.15:1. According to RDP4 analysis, the recombination observed in *mecA1* has been driven by recombination between different *S*. *sciuri mecA1* alleles (Supplementary S1B Fig). Although according to our data recombination in *mecA1* was not such a frequent event, the recombining *mecA1* alleles represented 60.5% of the *S*. *sciuri* population. In addition, all but two *S*. *sciuri* isolates showing oxacillin resistance carried recombining alleles, suggesting that recombination in *mecA1* was important for the development of resistance in this species.

**Fig 1:**
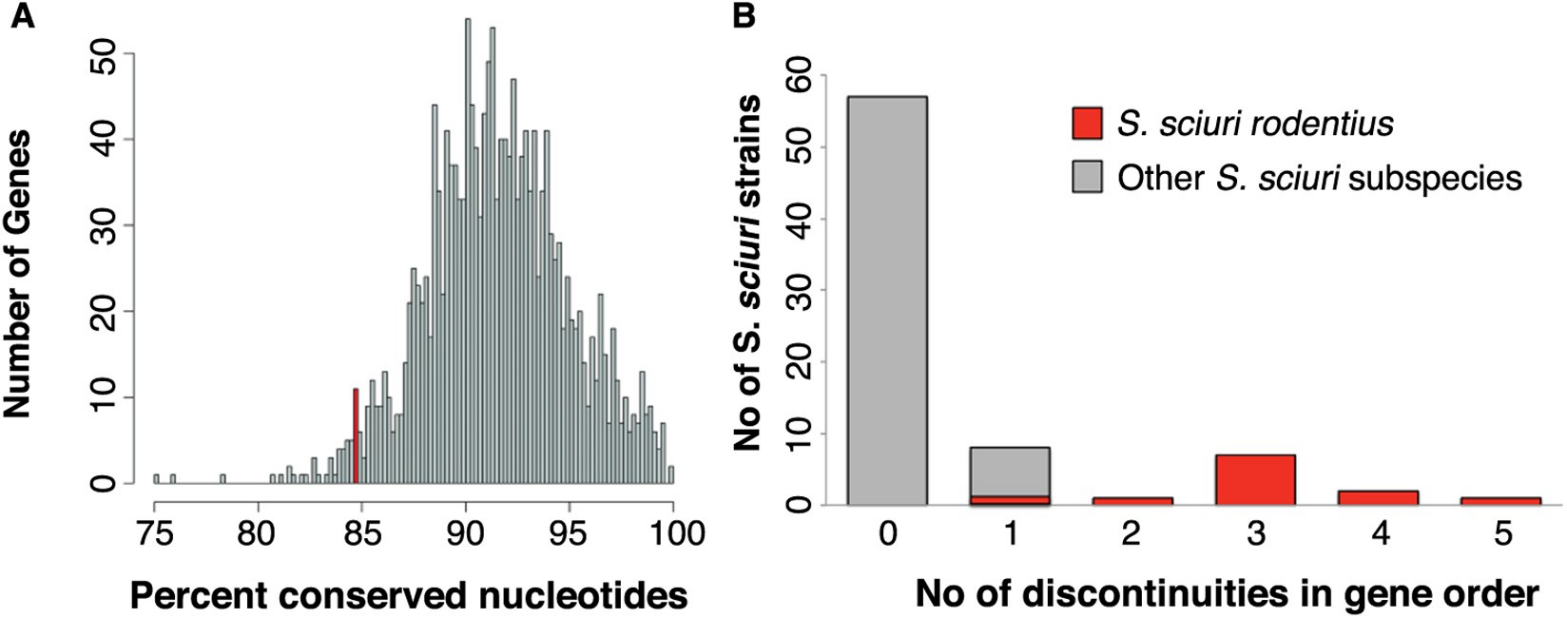
Distribution of the percentage of conserved nucleotides among the 1759 core genes of the 76 *S*. *sciuri* strains analyzed in this study. The conservation percentage of *mecA1* is shown in red (A). Number of discontinuities in the order of the core genes in the 76 *S*. *sciuri* strains analyzed in this study, when compared to *S*. *sciuri sciuri* reference strain NCTC12103 (B).

Overall, neither genetic diversity nor recombination were features affecting the entire *S*. *sciuri* genomes. This was obvious by the lower fraction of conserved positions of *mecA1* (84.7%) when compared to the remaining core genes (91.57%; stdev 3.43) (Fig 1A), and by the fact that *mecA1* was among the 2.5% most variable genes in the core genome (see supplementary S2 Table and Fig 1A). Additionally, for the great majority of strains (n = 57, 75%) the order of the 1759 core genes was conserved (0 discontinuities in gene order), when compared with the *S*. *sciuri sciuri* reference genome NCTC12103 (see Table 1 and Fig 1B).

**Table 1:** Number of discontinuities in the order of 1759 core genes of the 76 *S*. *sciuri* genomes when compared with the *S*. *sciuri sciuri* reference genome NCTC12103.

Discontinuities In Gene Order	0	1	2	3	4	5
Number of Genomes	57	8	1	7	2	1

The only subspecies that, according to our data, has probably a higher recombination rate is *S*. *sciuri rodentius*, since the genomes of all the strains belonging to this subspecies showed at least one discontinuity in their genome. Furthermore, this subspecies comprised the highest number of discontinuities in gene order (1–5 discontinuites). The other subspecies showing discontinuities were the subspecies *S*. *sciuri sciuri* and a putative new subspecies (see item *The genetic background was associated with the emergence of β-lactam resistance in S. sciuri*)), but this corresponded to a single discontinuity and was a rare occurrence among these subspecies.

Altogether, these results suggest that *mecA1* is a hotspot for diversification in *S*. *sciuri*.

### Different levels of oxacillin resistance were observed in all species of *S*. *sciuri* group

To assess the level of resistance to oxacillin, we determined the epidemiological cut-off (ECOFF) value for oxacillin in the *S*. *sciuri* group of species (S2 Fig), since the currently available MIC breakpoints are defined only for clinically significant *Staphylococcus* species. According to this analysis, the oxacillin breakpoint for resistance was set at 3 μg/ml oxacillin.

**Fig 2:**
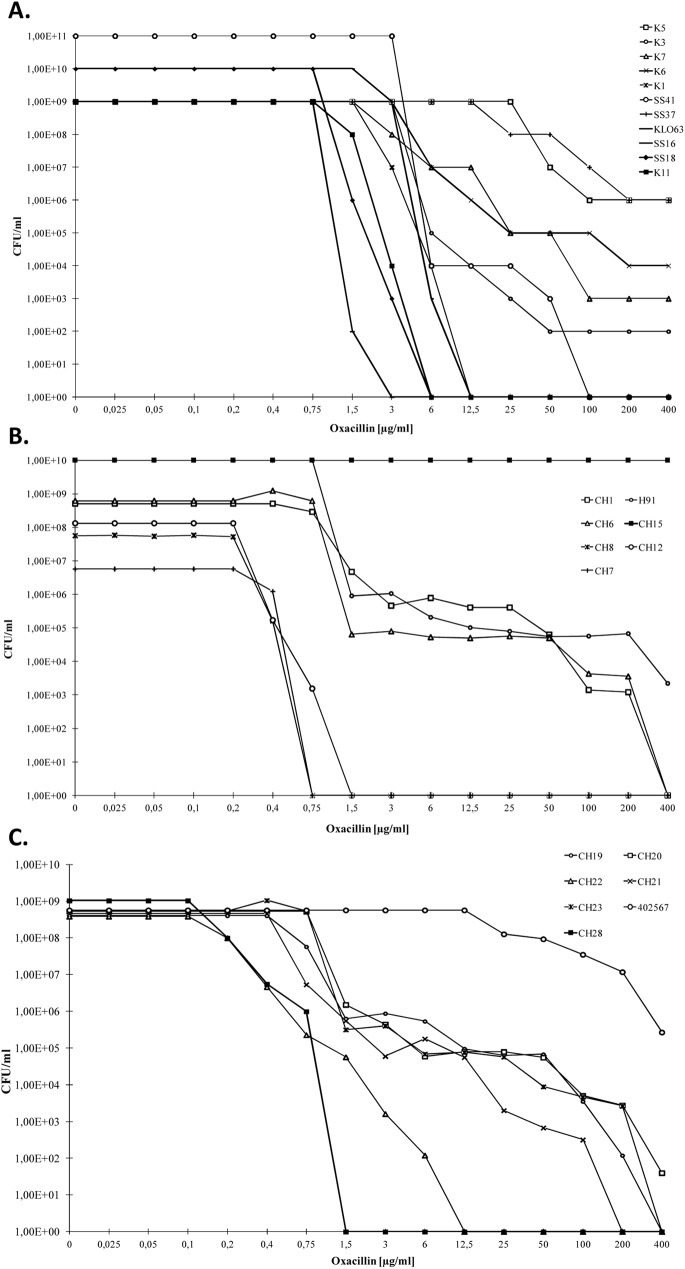
Oxacillin susceptibility population analysis profiles (PAPs) for representative *S*. *sciuri* (A)[15, 21], *S*. *vitulinus* (B) and *S*. *fleurettii* (C).

Considering this breakpoint, the great majority of *S*. *sciuri* strains carrying only *mecA1* (54/60) was susceptible to oxacillin, but six strains were resistant (K4, K5, K7, Jug17, SS37 and SS41) as determined by Etests. From the 54 susceptible strains, 24 produced heterogeneous profiles when analysed by oxacillin population analysis profiles (PAPs), and almost half of these isolates (11 out of 24) were able to grow at concentrations up to 6–100 μg/ml (Fig 2A). Moreover, the 16 *S*. *sciuri* strains that carried *mecA* in addition to *mecA1* were all resistant (MIC 16 to >256 μg/ml), and representative strains showed an heterogeneous profile and were able to survive at concentrations up to 800 μg/ml of oxacillin (Fig 2A and Table 2).

**Table 2:** Main characteristics of β-lactam resistant strains as defined by the ECCOF of 3 μg/mL.

Strain	Date of isolation	Host	MIC μg/ml (eTest/PAP)	Mechanism of resistance	Phylogenetic group	*mec* allele	Recombinant *mecA1* allele
M1234	2009	Human	>256	SCC*mec*	*S*. *sciuri* new 2	*mecA* 9 /*mecA1* 6	+
M692	2007	Human	96	SCC*mec*	*S*. *sciuri* new 2	*mecA* 9 /*mecA1* 6	+
M2590	2012	Human	>256	SCC*mec*	*S*. *sciuri* new 2	*mecA* 9 /*mecA1* 6	+
M2276	2011	Human	>256	SCC*mec*	*S*. *sciuri* new 2	*mecA* 9 /*mecA1* 6	+
D573	2007	Human	>256	SCC*mec*	*S*. *sciuri* new 2	*mecA* 9 /*mecA1* 6	+
M1653	2010	Human	>256	SCC*mec*	*S*. *sciuri* new 2	*mecA* 9 /*mecA1* 6	+
CH17	2010	Horse	>256	SCC*mec*	*S*. *sciuri* new 2	*mecA* 8 /*mecA1* 7	+
CH18	2010	Horse	>256	SCC*mec*	*S*. *sciuri* new 2	*mecA* 8 /*mecA1* 7	+
M2710	2012	Human	>256	SCC*mec*, altered PBP4	*S*. *sciuri* new 2	*mecA* 7 /*mecA1 4*	+
HSM851	2010	Human	16	SCC*mec*, altered PBP4	*S*. *sciuri* new 2	*mecA* 7 /*mecA1 4*	+
Jug17	2002	Human	>256	Altered PBP4	*S*. *sciuri* new 2	*mecA1* 4	+
K3	1992	Human	>256	SCC*mec/* alterations *mecA1* promoter	*S*. *sciuri rodentius*	*mecA*10/*mecA1* 17	+
K4	1992	Human	>256	Alterations *mecA1* promoter	*S*. *sciuri rodentius*	*mecA1* 17	+
K5	1992	Human	25	Alterations *mecA1* promoter	*S*. *sciuri rodentius*	*mecA1* 17	+
K7	1992	Human	>256	Alterations *mecA1* promoter	*S*. *sciuri rodentius*	*mecA1* 17	+
SS37	1996	Human	25	Alterations *mecA1* promoter	*S*. *sciuri rodentius*	*mecA1* 17	+
SS41	1996	Human	3	Alterations *mecA1* promoter	*S*. *sciuri rodentius*	*mecA1* 17	+
CH16	2010	Human	24	SCC*mec*	*S*. *sciuri rodentius*	*mecA* 7 /*mecA1* 19	+
K6	1992	Human	>256	SCC*mec*	*S*. *sciuri rodentius*	*mecA* 7 /*mecA1 42*	-
M1640	2010	Human	96	SCC*mec*	*S*. *sciuri sciuri*	*mecA* 7 /*mecA1 37*	-
Jug1	2002	Dog	>256	SCC*mec*	*S*. *sciuri* new 1	*mecA* 7 /*mecA1 16*	+
M1886	2011	Human	64	SCC*mec*	*S*. *sciuri* new 1	*mecA* 7 /*mecA1 43*	-
CH2	2004	Horse	4	Genetic background?	*S*. *vitulinus*	*mecA 4*	-
CH5	2005	Horse	>256	Genetic background?	*S*. *vitulinus*	*mecA* 4	-
CH15	2004	Horse	>256	alterations *mecA2* promoter	*S*. *vitulinus*	*mecA2* 2	-
CH19	2010	Horse	8	*mecA* native location	*S*. *fleurettii*	*mecA* 1	-
CH20	2010	Horse	6	*mecA* native location	*S*. *fleurettii*	*mecA* 3	-
CH21	2010	Horse	4	*mecA* native location	*S*. *fleurettii*	*mecA* 2	-
CH23	2010	Horse	4	*mecA* native location	*S*. *fleurettii*	*mecA* 2	-
CH24	2010	Horse	>256	*mecA* native location	*S*. *fleurettii*	*mecA* 2	-
CH25	2010	Horse	4	*mecA* native location	*S*. *fleurettii*	*mecA* 6	-
CH26	2010	Horse	>256	*mecA* native location	*S*. *fleurettii*	*mecA* 2	-
CH27	2010	Horse	4	*mecA* native location	*S*. *fleurettii*	*mecA* 2	-
CH29	2010	Horse	4	*mecA* native location	*S*. *fleurettii*	*mecA* 2	-
402567	2004	Horse	>256	*mecA* native location	*S*. *fleurettii*	*mecA* 5	-

Like in *S*. *sciuri*, in *S*. *vitulinus*, the great majority of strains carrying either *mecA2* or *mecA* were oxacillin-susceptible, but some of these strains showed a heterogeneous profile in which sub-populations could grow above the MIC (100–400 μg/ml) (Fig 2B). Moreover, a few strains displayed a resistant phenotype (CH15, CH2 and CH5) (see Table 2 and Fig 2B).

In contrast, the great majority of *S*. *fleurettii* isolates were resistant to oxacillin (MIC 4->256 μg/ml) with subpopulations that were able to grow at concentrations up to 25–400 μg/ml (Fig 2C), but two strains showed a susceptible phenotype (CH22 and CH28) (see Table 2 and Fig 2C).

### Oxacillin resistance emerged by multiple molecular strategies

In order to understand the mechanisms associated with the oxacillin resistance phenotypes exhibited by *S*. *sciuri*, *S*. *vitulinus* and *S*. *fleurettii* we looked for: differences in the structure of proteins encoded by *mecA* homologues; changes in the expression of *mecA* homologues; presence of SCC*mec*; and differences in the genetic background.

#### *Alteration in the structure of the active site in mecA homologue-encoded PBPs* was associated with β-lactam resistance

To test if the nucleotide diversity of different *mecA* homologues could explain the different levels of oxacillin susceptibility observed, we compared the structure of the proteins encoded by different *mecA* homologues (*mecA* allele 5, *mecA2* allele 2, *mecA1* allele 4, *mecA1* allele 17, *mecA1* allele 21, *mecA1* allele 22, *mecA1* allele 25 and *mecA1* allele 42) with the structure of PBP2a using a modeling approach. We observed that in multiple occasions, in *S*. *sciuri*, *S*. *vitulinus* and S. *fleurettii*, the level of oxacillin susceptibility could be related to alterations occurring in the active site grove that could lead to a higher or lower exposure of the catalytic amino acid Ser403 (PBP2a)/Ser401 (PBP4).

**Fig 3:**
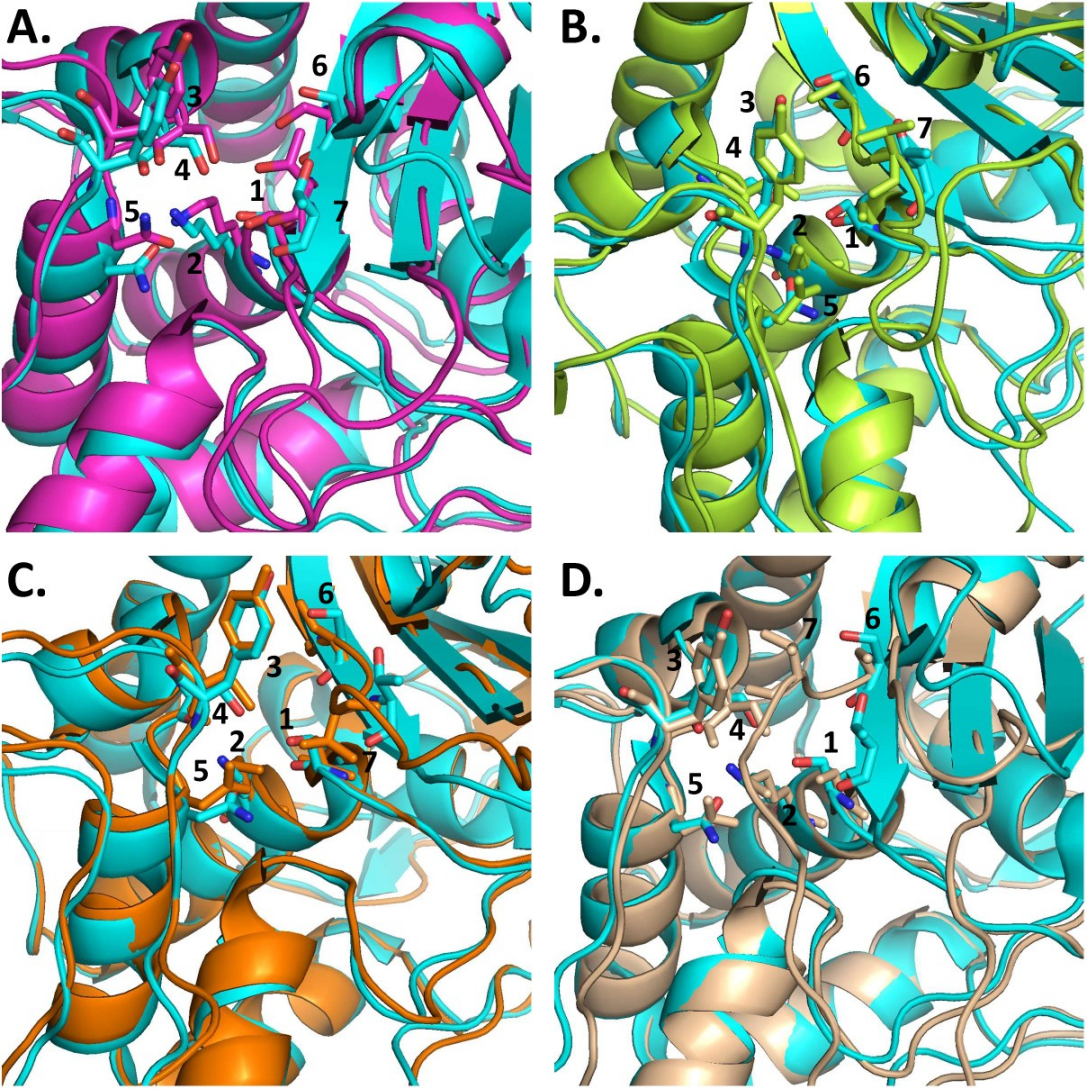
Alignment of the active centre of PBP2a (highlighted in cyan blue) and representative PBPs putatively encoded by *mecA* homologues. The structure of the PBP was predicted by Modeller and the alignment was produced in Pymol. Oxacillin-resistant *S*. *fleurettii* 402567 *mecA* allele 5/PBP2a (A). Oxacillin-susceptible *S*. *vitulinus* CH10 *mecA* allele 2/PBP2a (B). Oxacillin-susceptible *S*. *sciuri* K11 *mecA1* allele 42/PBP2a.1 (C). Oxacillin-resistant *S*. *sciuri* JUG17 *mecA1* allele 4/PBP2a (D). 1. Ser401/Ser403. 2. Lys404/Lys406. 3. Tyr444/Tyr446. 4. Ser460/Ser462. 5. Asn464/Asn466. 6. Ser596/Ser598. 7. Thr598/Thr600.

This was the case of the protein encoded by *mecA1* allele 42, present in the oxacillin-susceptible *S*. *sciuri carnaticus* type strain K11 [22], in which the residues Ser596 and Thr598, were not located in a β-sheet motif, in contrast to what was found in PBP2a (Fig 3C). The position of these residues, which surround the active site groove, was otherwise more relaxed, suggesting that access to Ser401 is probably facilitated, explaining the susceptible phenotype observed. The structures predicted for the remaining *mecA1* alleles analyzed were similar. This association between the phenotype and the protein structure was likewise found in the *S*. *sciuri* strains Jug17, HSM851 and M2710, carrying allele *mecA1* 4 (Fig 3D), all showing high-level resistance to oxacillin, in which Thr598 was much closer to Tyr444, thus “closing” the active site groove and protecting Ser401 from interacting with the β-lactam ring.

In addition, the protein encoded by the most frequent *mecA2* allele (*mecA2 2*) in *S*. *vitulinus*, (which is associated with oxacillin susceptibility in almost all strains), had residues Ser403 and Thr600 (Fig 3B) in positions different from those found in PBP2a. This could lead to higher exposure of Ser403 to the β-lactam ring. The only strain carrying *mecA2* allele 2 that was resistant to oxacillin (CH15) had an alteration in the promoter of the protein (see section below—*Mutations in the promoter of mecA homologues gave rise to β-lactams resistance*).

Another example, in *S*. *fleurettii*, is the protein encoded by *mecA* allele 5, associated with high level of oxacillin resistance that had the exact same residues as *S*. *aureus* PBP2a. Although these residues were not in the exact same position, their orientation was the same (Fig 3A), suggesting that they should have a similar rate of acylation.

#### Mutations in the promoter of mecA homologues gave rise to β-lactam resistance

Genetic changes in the *mecA1* promoter of *S*. *sciuri* strains SS37 and SS41 –namely the insertion of IS*256* and a single-nucleotide polymorphism (SNP)–respectively, were previously reported by Couto *et al*. to be associated with a resistance phenotype [16]. In our collection, we additionally identified several cases of alterations in the promoter region (200 bp upstream of *mecA* homologues) that were associated both with the expression level of the PBPs and to the oxacillin susceptibility profile observed.

For instance, the oxacillin-resistant *S*. *sciuri rodentius* strains K4, K5 and K7 showed alterations in -10 and -35 sequences when compared to the susceptible strain K11 (see S3 Fig). In particular, we were able to identify in these strains the same nucleotide alterations previously reported for the oxacillin resistant SS41 strain [16]. For *S*. *vitulinus*, the promoter regions of two strains carrying the *mecA2* allele 2 –one susceptible (CH10) and the other resistant to oxacillin (CH15)–were compared. Whereas the ribosome binding site (RBS) sequence, GGGAGGG, was located immediately upstream of *mecA2* in strain CH15 (at position -3), this same sequence was located further upstream (at position -6) in strain CH10. Finally, for *S*. *fleurettii*, the promoter of the susceptible strain (CH28) showed a deletion of 16 bp at position -29 upstream of *mecA*, possibly at the -10 region (TATACT), when compared to strain CH22, which expresses a higher level of heteroresistance (see Fig 2C).

**Fig 4:**
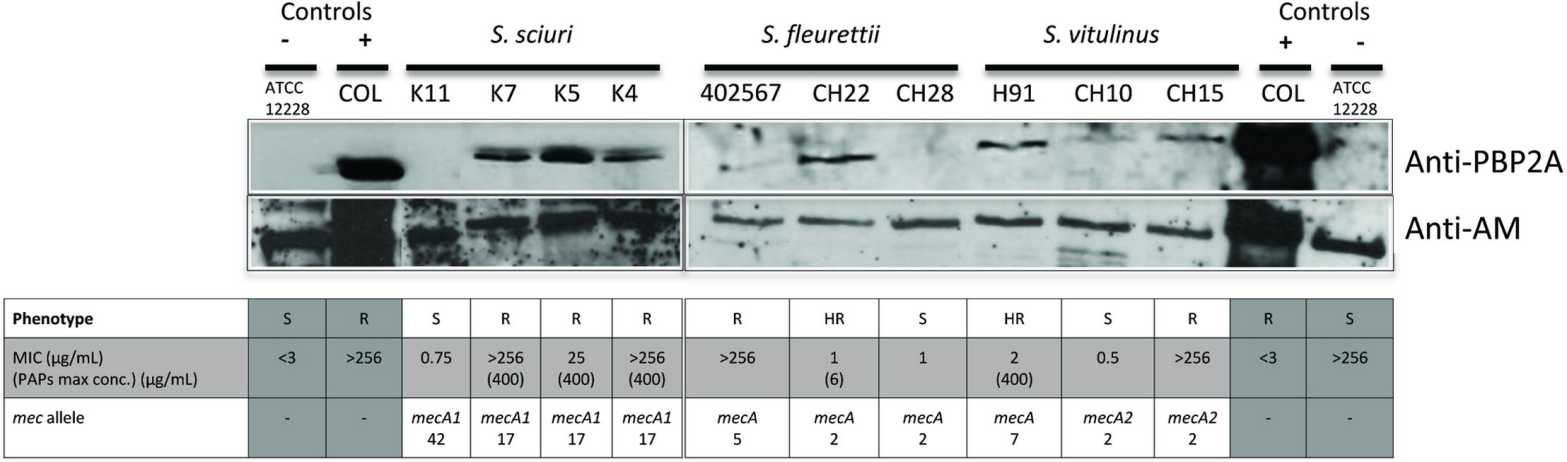
Western blotting of the membrane fraction of *S*. *sciuri*, *S*. *fleurettii* and *S*. *vitulinus* using a polyclonal antibody raised against *S*. *aureus* PBP2a and a polyclonal antibody raised against the amidase (AM) domain of *S*. *aureus* Atl protein. Lanes 1 and 14: *S*. *epidermidis* strain ATCC12228; Lanes 2 and 13: *S*. *aureus* strain COL; Lane 3: *S*. *sciuri carnaticus* strain K11; 4: *S*. *sciuri rodentius* strain K7; 5: *S*. *sciuri rodentius* strain K5; 6: *S*. *sciuri rodentius* strain K4; 7: *S*. *fleurettii* strain 402567; 8: *S*. *fleurettii* strain CH22; 9: *S*. *fleurettii* strain CH28; 10: *S*. *vitulinus* strain H91; 11: *S*. *vitulinus* strain CH10; 12: *S*. *vitulinus* strain CH15.

As shown in Fig 4, all the described alterations in the promoter sequence could be associated with differences in the expression of the *mecA* homologues encoded proteins. In particular, expression of *mecA1* in resistant *S*. *sciuri* strains K4, K5, K7 and of *mecA2* in *S*. *vitulinus* strain CH15 was increased when compared to the susceptible strains K11 and CH10. The expression of *mecA* in the *S*. *fleurettii* susceptible strain CH28 was decreased when compared to the heterogenous strain CH22.

The association between nucleotide alterations in the sequence of the *mecA* homologues and their respective promoter regions and the resistant phenotype to oxacillin, was found to be statistically significant (p<0.05), suggesting that these associations were not due to chance.

Other factors that are described to influence *mecA* expression in *S*. *aureus* are the regulatory systems *mecI*/*mecR1* and *blaI*/*blaR1* that are induced in the presence of antibiotic [23, 24]. The *mecA* was the only *mec* homologue in which *mecI*/*mecR1* were found upstream the gene and although a *blaZ* homologue was found in the three species (67% aminoacid sequence identity), it seems not to have the capacity to hydrolyze the β-lactam ring as observed using the nitrocefin test. Moreover, no difference in the expression of *mec* homologues was observed in the presence of oxacillin by western blotting, which suggests that these regulators in species of the *S*. *sciuri* group do not respond to the same stimulus as in *S*. *aureus*.

#### The presence of SCC*mec* is associated with β-lactam resistance only in *S*. *sciuri*

The main mechanism of β-lactam resistance is associated with the presence of SCC*mec* [1]. This element occurred in 20% of all *S*. *sciuri* analyzed and in all subspecies except *S*. *sciuri carnaticus* (see Table 2) and was always associated with high-level resistance (16<MIC<256) (see Table 2). Although in SCC*mec* carrying strains, *mecA1* was also present, the majority of *mecA1* alleles (*mecA1 6*, *mecA1 7*, *mecA1 8*, *mecA1 37*, *mecA1 42*) and promoters found in these strains were carried by oxacillin-susceptible strains too, suggesting that SCC*mec* and not *mecA1* is responsible for the resistance phenotype (see supplemental S1 Table). Three strains carried both SCC*mec* and alterations in *mecA1* structure (HSM851 and M2710) or promoter (K3), which were identified above as being possibly associated with resistance. In these cases both mechanisms may be contributing to the resistance phenotype.

In the other two species SCC*mec* was either absent (*S*. *fleurettii*) or did not confer resistance to oxacillin (*S*. *vitulinus*) (see Table 2 and S3B Fig). The absence of the resistance phenotype, in *S*. *vitulinus*, in spite of the presence of SCC*mec* is puzzling. However, this is not related to the lack of *mecA* expression since, as shown by Western blotting, strain H91, carrying SCC*mec*, showed an expression level of the encoded PBP that was higher than the negative control ATCC12228.

#### The genetic background was associated with the emergence of β-lactam resistant phenotype in *S*. *sciuri*

The contribution of several housekeeping genes to the optimal expression of methicillin resistance was previously described in *S*. *aureus* [11, 14], and evidence has been presented that not all *S*. *aureus* lineages are adapted to express methicillin-resistance [25, 26].

The phylogenetic reconstruction based on the number of SNP differences in the core genome of species of the *S*. *sciuri* group revealed the existence of three well-defined phylogenetic groups, corresponding to each of the three species. While the core genomes of *S*. *fleurettii* and *S*. *vitulinus* had an average 3000–9000 SNPs and 4000–7000 SNPs difference, respectively, *S*. *sciuri* core genomes differed in average by 15000 SNPs, (S3A–S3C Fig). Moreover, within *S*. *sciuri* species five different clusters were identified, that probably correspond to five different subspecies: the three previously described subspecies (*S*. *sciuri sciuri*, *S*. *sciuri rodentius* and *S*. *sciuri carnaticus*) and two putative new subspecies. The phylogenetic clustering shown might have been influenced by recombination events occurring in *S*. *sciuri rodentius* (see above). However, with the exception of one strain (K4), all the *S*. *sciuri rodentius* strains identified by other methods, like *tuf* and 16S rDNA sequencing, clustered together in the same large subspecies branch, suggesting that genetic recombination must have mainly altered the phylogenetic relationships at the strain level only, not altering significantly the definition of the subspecies.

**Fig 5:**
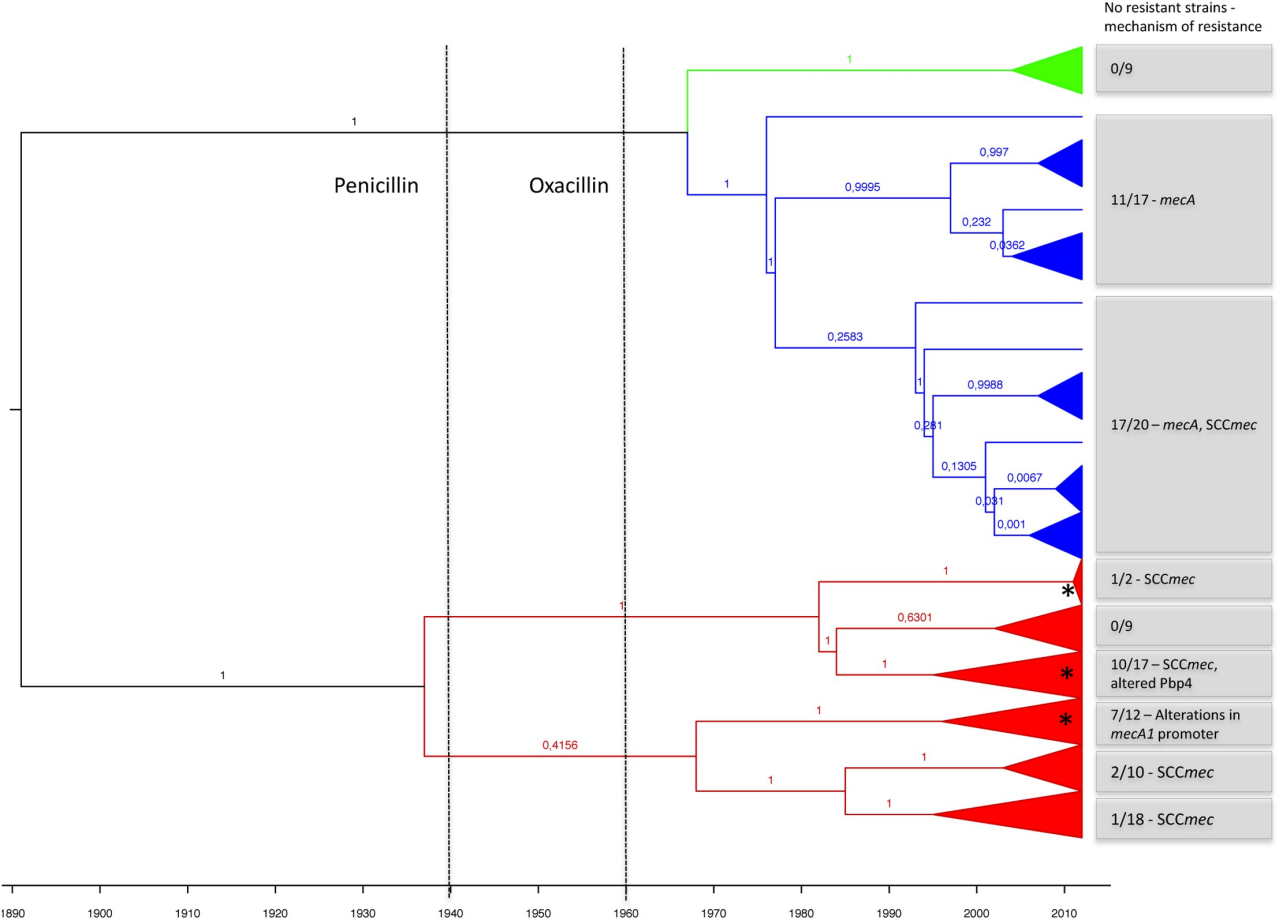
Evolutionary history of *mecA* homologue alleles. BEAST analysis of the nucleotide sequence of *mecA* homologues using the random clock and constant population models. *mecA1* alleles are shown in red, *mecA* alleles are shown in marine blue and *mecA2* alleles are shown in green. Numbers next to tree branches are the posteriors for the tree. Dashed lines indicate the time of introduction of penicillin and oxacillin into clinical practice in humans. Grey boxes include the number of resistant isolates within each branch and the associated mechanisms of resistance. Asterisks indicate the clusters in which recombinant *mecA1* alleles were identified.

The resistance phenotypes were clearly associated with phylogeny, mainly in *S*. *sciuri*. The isolates showing β-lactam resistance were almost exclusively (19/22) confined to *S*. *sciuri rodentius* and *S*. *sciuri subspecies new 2* and to specific clusters within each of these subspecies, which were distinct from the clusters containing susceptible isolates (p<0.05) (See S3C Fig and Fig 5). Moreover, resistant strains were distributed along phylogeny according to the mechanism of resistance involved. While strains showing alterations in promoter were exclusively associated with the subspecies *S*. *sciuri rodentius*, those showing alterations in the structure of the proteins encoded by the *mec* homologue or having SCC*mec* structures belonged mainly (14/17) to the putative *subspecies new 2* (Table 2) (p<0.05). These results suggest that the genetic background was a key factor for the expression of resistance in *S*. *sciuri*. However, the identification of the genetic background factors contributing to resistance were not further explored.

In contrast to *S*. *sciuri*, in which emergence of β-lactam resistance appears to be strongly associated with phylogeny, strains of *S*. *vitulinus* and *S*. *fleurettii*, with very few exceptions, were uniformly susceptible and resistant to β-lactams, respectively (see supplementary S1 Table). However, in these species we could also find examples that illustrate the impact of the genetic background in β-lactam resistance. In particular, we found that *S*. *fleurettii* strains carrying the exact same gene allele as susceptible *S*. *vitulinus* (like *mecA* allele 4 and *mecA* allele 2) showed in contrast, high-level of resistance to β-lactams.

### Human and human created environments were drivers of β-lactam resistance

*S*. *sciuri* is widely disseminated in nature, being found in different animal species and, occasionally, isolated from human infections, whereas *S*. *vitulinus* and *S*. *fleurettii* host range is mainly restricted to animals (36–39). Nonetheless, the three species live in environments created by humans in which antibiotic usage is frequent, namely in hospitals and farms.

Data resulting from this study provided evidence that antibiotic deployment in these environments were probably the drivers of β-lactam resistance development. This is illustrated by the finding that all β-lactam resistant *S*. *sciuri*, were collected from human sources or from animals in close contact with humans (horses and dogs), and not from wild animals (p<0.05). Moreover, all *S*. *vitulinus* and *S*. *fleurettii* included in this study originated from animal species in close contact with humans and were either intrinsically resistant to β-lactams or had the capacity to develop resistance.

### Antibiotic usage is linked to the emergence of β-lactam resistance

Antibiotic resistance is believed to be the result of antibiotic pressure imposed on bacteria. Bayesian phylogenetic reconstruction was used to explore the association between the time of emergence of resistant phenotypes and antibiotic use.

The MCC tree resulting from BEAST analysis of *mecA* homologs using a random molecular clock and a constant size population model showed that the time to the most recent common ancestor (tMRCA) of all *mecA* homologues alleles was estimated to be in 1891 (1845–1976 95%HPD) (see Fig 5), before the introduction of antibiotics in the clinical practice, and that two clusters split in 1937 (1892–1968 95%HPD), one originating *mecA1* and the other *mecA2* and *mecA* (Fig 5 and S1B Fig). The *mecA1* began diversifying in 1968 (1951–1986 95%HPD), which coincided with the emergence of recombining alleles and the emergence of β-lactam resistance. This was contemporaneous with the use of penicillin and methicillin as a treatment in humans (1940 and 1960, respectively) and of penicillin as a feed additive in production animals (1950–1960).

According to our results, the first *mecA2* allele emerged in a *S*. *vitulinus* strain, approximately in 1967 (1930–1976 95%HPD). The development of *mecA* occurred later, in *S*. *fleurettii*, in 1977 (1961–1997 95%HPD), suggesting *mecA* could have been already present in the population when methicillin was first introduced into clinical practice, in 1961. The SCC*mec* was estimated to have emerged afterwards (1982–1994 95%HPD) in *S*. *sciuri rodentius* [22] (Fig 5). Once created, SCC*mec* appears to have been rapidly disseminated to other staphylococcal species like *S*. *aureus*; coincidently, it was during the 1980s and 1990s that MRSA pandemic clones began to expand worldwide.

The 95% HPD values obtained for the dates presented are wide, particularly at deeper nodes, thus comprising considerable uncertainty. This may result from the type of strain collection analyzed, which was non-random and enriched for more recent isolates. Alternatively, the findings may reflect occurrence of purifying selection and recombination in *mecA1*. In particular, leaps of diversity due to recombination in *mecA1* or to a weak/mild purifying selection may have led to the estimation of a date that is posterior to the true date of emergence. However, the MCC tree based on *mecA* genes in the absence of recombination sites (and using the same molecular clock and population models), showed no relevant differences when compared to the tree constructed in the presence of recombination, neither in the population structure nor in the dating of evolutionary events.

## Discussion

The mechanism of β-lactam resistance mediated by *mecA* in *Staphylococcus* is one of the most efficient mechanisms of resistance to antibiotics, providing resistance to virtually all members of the large class of β-lactams. Several studies have shown that the *mecA* precursor was a native gene (*mecA1*) not providing resistance in *Staphylococcus sciuri*, the most primitive staphylococcal species [15, 18]. However, the evolutionary steps leading to phenotypic resistance remained unclear. In this study, we showed that species of the *S*. *sciuri* group developed multiple strategies during their evolutionary history to develop β-lactam resistance including (i) structural diversification of a native PBP, (ii) changes in the promoter of the *mecA* homologues, (iii) SCC*mec* acquisition and (iv) adaptation of the genetic background.

Although the TP domain has been described as the crucial domain for PBP activity [7], our results are the first to identify a fundamental role of the NB domain for its full performance. In particular, we found that alterations in the NB domain of proteins encoded by the *mecA* homologues can have impact on the level of distortion of the active site groove and on the consequent access of the substrate (or the antibiotic) to the Ser401/Ser403, the key aminoacid residues at the catalytic site. The existence of subtle changes in the NB domain of PPBs can give rise to proteins with different levels of activity. Since the different *mecA* homologues have a very conserved TP domain, the evolution from a susceptible to a resistance determinant probably involved alterations mainly in the NB domain of the protein.

An additional mechanism driving β-lactam resistance involved alterations in the promoter of *mecA* homologues: either deletions around the RBS site or alterations in -10 and -35 regions. The association of changes in the promoter with an increased *mecA1* expression and a resultant resistance phenotype was a phenomenon previously observed in a few strains of *S*. *sciuri* [16]. In this study we confirmed that these type of events probably occurred with a relatively high frequency in the overall *S*. *sciuri* population and also in *S*. *vitulinus*, during their evolutionary history. This event occurred in the promoters of *mecA1* and *mecA2* only, and may represent a molecular strategy used by the bacteria to circumvent antibiotic pressure, in the absence of a low affinity PBP. We found a good correlation between the alterations in the promoter of these genes and both the expression of their encoded proteins as well as the corresponding resistance phenotypes. A previous study analyzing the mechanism of β-lactam resistance in *S*. *sciuri* strains showed a total correlation of the resistance phenotype with an increase in both *mecA1* transcription and *mecA1*-encoded PBP translation (16), suggesting that post-transcriptional and post-translational regulation, if occurring, appear not to have disturbed the link between the alterations in the promoter and the observed expression of resistance.

Another event associated with the emergence of resistance was the acquisition of SCC*mec* by *S*. *sciuri* and *S*. *vitulinus*. However, in this case the correlation between the phenotype and the genotype was only observed for *S*. *sciuri*. The absence of a resistant phenotype in *S*. *vitulinus* strains carrying *mecA* either in the native location or within SCC*mec* is puzzling. We show that the susceptibility is not associated with the absence of gene expression, but post-translational modifications may be involved. Another alternative is that access of the antibiotic to its target may be blocked, by an unknown mechanism.

In addition, our results demonstrate that–similarly to the case of MRSA [11, 14, 27]–the genetic background also plays an important role in the expression of β-lactam resistant phenotype of this primitive group of staphylococci. The most obvious examples are the absence of resistant phenotype in the presence of *mecA* in *S*. *vitulinus* and the development of resistance in particular phylogenetic clusters of *S*. *sciuri*. Genes involved in general metabolism were already shown to play important roles in the expression of β-lactam resistance in *S*. *aureus* suggesting an interplay between the overall metabolism and β-lactam resistance [27] [28].

The observation that unknown factors in genetic background are important for the expression of resistance does not allow to establishing definitely a direct correlation between nucleotide substitutions observed in the promoter sequence and *mec* homologue genes and the resistance phenotype. To substantiate this, an ideal approach would be to test the different promoters and express the different *mec* homologue variants in an appropriate *S*. *sciuri* genetic background. However, these studies are very difficult to perform due to the lack of genetic tools available in this species.

The fact that such diversified mechanisms leading to β-lactam resistance were found in different species of the *S*. *sciuri* group together with accumulation of more than one of these mechanisms at different time points, generating redundancy, are evidence for the persistent antibiotic pressure that these species experienced during their evolutionary history. Moreover, the diversification, recombination and purifying selection, observed in *mecA1* gene, in opposition to the remaining chromosome, in the majority of *S*. *sciuri* strains further highlights the specific response of a bacterial species to the environmental pressure by antibiotics.

Antibiotic pressure giving rise to β-lactam resistance appears to be directly linked to exposure to human created environments, since resistance was exclusively observed in clinical isolates of human origin or from production animals, where high doses of antibiotics are generally used, and absent from wild animals where antibiotic pressure is limited to the level of antibiotics present in nature. This is in accordance with the Bayesian analysis performed, in which the estimated dates of occurrence of key events in *mecA* homologues evolution coincided with the time of introduction of antibiotics in veterinary and human clinical settings [29].

A limitation of this study is the fact that the reconstructed phylogeny of the *mecA* homologues was based on the Bayesian analysis of genes, which we showed to be under recombination and purifying selection. Additionally, it was based on a sampling framework that was non-random and that constituted an underrepresentation of *S*. *sciuri*, *S*. *vitulinus* and *S*. *fleurettIi* population diversity, namely in host range, dates of isolation and geographic region [30]. Consequently, inexact estimations of the evolutionary path of *mecA* homologues, mainly of *mecA1*, may have been generated, adding uncertainty to the dating of the evolutionary events of *mecA* homologs, namely to the existence of overlap between the emergence of resistance phenotypes and the use of antibiotics.

Overall our data suggest that the first evolutionary steps leading to *mecA*-mediated β-lactam resistance in *Staphylococcus* occurred in the most primitive staphylococcal species by several molecular mechanisms, in response to β-lactam pressure, both in humans and livestock. These results highlight the complexity of the evolution of *mecA*-mediated β-lactam resistance.

## Methods

### Ethical Statement

Human isolates were obtained as part of routine surveillance and laboratory testing and were analyzed anonymously. All data was collected in accordance with the European Parliament and Council decision for the epidemiological surveillance and control of communicable disease in the European community [31, 32]. Ethical approval and informed consent were for that reason not required. The animal isolates originated from nasal and skin swabs and bovine milk and some were collected as part of previous studies in Denmark [33, 34] and Switzerland [19, 35]. According to the national legislations, formal ethical approval was not required since samples were collected by non-invasive sampling procedures and no animal tissues were collected.

### Bacterial strain collection

A collection of 106 staphylococcal isolates, comprising 76 *S*. *sciuri*, 18 *S*. *vitulinus* and 12 *S*. *fleurettii* was assembled. This is a convenience sample, however, we believe it reasonably reproduces the species distribution and diversity of hosts that exist in nature, as previously described [36–39].

Regarding *S*. *sciuri*, 28 isolates were obtained from humans, while the remaining 45 isolates were recovered from both wild and domesticated mammals (Supplementary S1 Table). Isolates were collected in different countries (Czech Republic, Denmark, Portugal, Switzerland, Sweden, former Yugoslavia, Mozambique, Panama and USA) between 1972 and 2012. *S*. *vitulinus* and *S*. *fleurettii* isolates were collected from horses and bovine mastitis milk samples, in Denmark, Switzerland and the Netherlands, in 2004, 2005 and 2010. The *S*. *sciuri* isolates were identified at the species level by 16S rRNA ribotyping and API-Staph (Biomerieux, France). *S*. *fleurettii* and *S*. *vitulinus* were identified at the species level by sequencing of 16S rRNA or *sodA* and Maldi-tof analysis (Microflex LT, Bruker Daltonics GmbH, Bremen) [19, 35, 40]. Species identification was confirmed by phylogenetic analysis of *tuf* gene nucleotide sequence [41].

### β-lactam susceptibility

Was assessed by oxacillin Etest (bioMérieux, France). The breakpoint for defining susceptibility was evaluated as suggested by EUCAST (www.eucast.org). An epidemiological cut-off value (ECOFF) was determined by considering the MIC to oxacillin of *S*. *sciuri* and *S*. *vitulinus* isolates not carrying *mecA* (wild type) and isolates carrying *mecA* (non-wild type; resistant). All *S*. *fleurettii* strains carried *mecA* and were therefore considered resistant. The distribution of MIC values was plotted (S2 Fig) and isolates were considered susceptible when MIC < 3 μg/ml. Moreover, population analysis profiles (PAPs) for oxacillin were determined for representative isolates (28/60 *S*. *sciuri* exclusively carrying *mecA1*, 23/37 isolates carrying *mecA*, 9/9 isolates carrying *mecA2*) as previously described [42]. The PAP results of *S*. *sciuri* isolates have already been published [15, 21].

### Whole-genome sequencing and *de novo* assembly

DNA was extracted using the phenol/chloroform extraction method (*S*. *sciuri*) and the DNEasy Blood & Tissue Kit (*S*. *vitulinus* and *S*. *fleurettii*) (Qiagen, Limburg, The Netherlands). Sequencing was performed using a HiSeq (Illumina, San Diego, USA) with an estimated coverage of 40x and a read length of 100 bp. The reads were assembled *de novo* using VELVET [43] and VelvetOptimiser (https://github.com/Victorian-Bioinformatics-Consortium/VelvetOptimiser.git).

### Reference genome *S*. *fleurettii* 402567

DNA of *S*. *fleurettii* 402567 was prepared by phenol/chloroform extraction and was sequenced using PacBio RS apparatus (Pacific Biosciences, Menlo Park, USA). De novo assembly was performed using HGAP 3 (https://github.com/PacificBiosciences/Bioinformatics-Training/wiki/HGAP-in-SMRT-Analysis).

A reference genome was produced by combining Illumina and PacBio sequencing data for a single strain, *S*. *fleurettii* isolate 402567. PacBio reads were combined with Illumina reads obtained for each isolate in CLC Genomics Workbench (Qiagen, Hilden, Germany), using the Genome Finishing module. The resulting contigs were ordered using the closed genome of *Staphylococcus xylosus*, the species most closely related to *S*. *fleurettii* with a closed genome (NCBI accession number CP007208.1; average nucleotide identity with *S*. *sciuri*, 78%; *S*. *vitulinus*, 77.1%; and *S*. *fleurettii*, 78.5%). Gaps (eight) were closed by mapping Illumina data of remaining *S*. *fleurettii* strains to the contigs. The resulting closed genome was annotated with RAST (http://rast.nmpdr.org/).

### Estimation of strain-to-strain phylogenies

The reference genome *S*. *fleurettii* 402567 was used to perform a SNP analysis of the predicted core genome of *S*. *sciuri*, *S*. *vitulinus* and *S*. *fleurettii* isolates. SNP analysis was performed using Stampy (version 1.0.11) where reads were mapped to the reference genome. SNP calling was performed using SAMtools (version 0.1.12), and Neighbor Joining (NJ) analysis was used to assess the phylogeny. Trees were drawn using FigTree (http://tree.bio.ed.ac.uk/software/figtree/).

### Phylogenetic analysis of *mecA* homologues

Nucleotide sequences of *mecA* homologues were identified by BLAST analysis and were extracted from the sequence of the contigs. Alignments with the entire gene or regions corresponding to specific domains were performed with ClustalW [44]. Phylogenetic trees were constructed with a neighbor-joining algorithm. We used BEAST software (v1.8.3) [45] to investigate the temporal evolution of *mecA* homologues. Estimation of substitution rates and divergence times of the tree internal nodes was performed using the HKY nucleotide substitution model. The Markov chain Monte Carlo (MCMC) analysis was run up to 10^7^ generations and checked for convergence by examining that the effective sample size (ESS) values were greater than 200 for all parameters. Strict, random, uncorrelated and fixed clock models under a constant population size model were compared for their fit to the data using marginal likelihood (stepping stone and path sampling) (see S3 Table) and Bayes factor (see S4 Table). The best-fit clock model (random clock) was then tested with the constant size population and the exponential growth population models. No significant differences in timescales or tree topology were obtained when the two different population size models were used. A burn-in of 10% was removed of each BEAST run and the maximum clade credibility (MCC) tree was selected from the posterior tree distribution using the program TreeAnnotator (available as part of the BEAST package). Final trees were annotated with FigTree (http://tree.bio.ed.ac.uk/software/figtree/). The BEAST analysis was performed for the entire set of *mec* homologue sequences and for the sequences belonging to each *mec* gene separately. To assess the impact of recombination on the phylogeny, the same analysis was repeated with the *mec* gene homologue sequences from which nucleotides under recombination were deleted (RDP4 software).

### Estimation of dN/dS ratios

To verify if the non-binding and the transpeptidase domain of *mecA1* were under positive selection, estimates of overall dN/dS ratios (number of non-synonymous substitutios per site/number of synonymous substitutions per site) were produced for the nucleotide sequence of each domain, using the program MEGA6 [46].

### Estimation of recombination/mutation rates

RDP4 [47] was used to predict which parts of the *mecA* homologue sequences were under recombination and to estimate the *mecA1* recombination/mutation rate.

### Modeling of protein structure

The structure of representative proteins encoded by the *mecA* homologues was predicted using ModWeb (https://modbase.compbio.ucsf.edu/modweb/) [48]. Structures of one *mecA* allele, one *mecA2* allele and six *mecA1* alleles (representing each major clade of the phylogenetic tree, 0.015 distance cut off) were obtained. Alignments of the structures modeled with PBP2a (protein database, PDB code 1MWU) were produced in PyMol (The PyMOL Molecular Graphics System, Version 1.5.0.3 Schrödinger, LLC) and visually inspected for relevant alterations of the protein structure.

### Assessment of genetic diversity and synteny of *S*. *sciuri* core genes

The genetic diversity of the different *mecA* homologues was assessed by the Simpson’s index of diversity (SID) [49], using a confidence interval of 95%. The online tool available at http://darwin.phyloviz.net/ComparingPartitions/ was used. To find the core genes of the 76 *S*. *sciuri* genomes, we used Prokka [50] and Roary [51]. The sequences of the aligned core genes were compared using Weblogo version 2.8.2 [52] and the number of conserved positions were determined for each core gene.

To determine the order of the 1759 core genes in each contig of the 76 *S*. *sciuri* assembled genomes a consensus sequence of each core gene was blasted against the 76 *S*. *sciuri* genomes and the reference genome NCTC12103 and their position assigned. The order of the core genes in each contig of the 76 genome sequences was compared to the order of the core genes in the NCTC12103 genome, and the total number of discontinuities was determined using an in-house script.

### Statistical analysis

Association between variables within the data was done using the Qui^2^ test with 95% confidence level.

### Purification of staphylococcal membrane fraction

*S*. *aureus* strain COL, *S*. *epidermidis* strain ATCC12228, *S*. *sciuri* strains K11, K7, K5 and K4, *S*. *fleurettii* strains 402567, CH22, CH28 and *S*. *vitulinus* strains H91, CH10 and CH15, were grown in 250 ml of TSB at 37°C with aeration to an OD_600nm_ of 0.7. Cells were harvested, washed and ressuspended in buffer A (50 mM Tris pH 7.5, 150 mM NaCl, 5 mM MgCl_2_) with phenylmethylsulfonyl fluoride (0.5 mM) and submitted to freeze-thaw cycles. All subsequent steps were performed at 4°C. Lysostaphin (100 μg/ml), Lysozyme (50 μg/ml), DNase (10 μg/ml), RNase (10 μg/ml), PMSF (0.5 mM) β-mercaptoethanol (10 mM) were added and the cell suspensions were incubated on ice for 30 min, followed by 5 cycles of sonication of 30 sec and 2 min intervals. Unbroken cells and cellular debris were removed by centrifugation of 5 min at 5000 *g* and the resulting supernatants were centrifuged at 50,000 *g* for 1h and washed in 50 mM phosphate buffer, pH 7.0. The obtained membrane fraction was resuspended in 25mM phosphate buffer pH 7.0, 1% Triton X-100, 10 mM MgCl_2_, 20% glycerol. Total protein concentration was determined using the BCA assay (Pierce, Thermo Scientific, USA).

### Detection of PBP2a by western blotting

Membrane preparations (50 μg) were separated by SDS polyacrylamide gel electrophoresis (8% acrylamide-0.06% bisacrylamide) at constant current of 20 mA. The proteins were transferred onto nitrocellulose Hybond-ECL membranes (GE Healthcare Life Sciences, USA) using the wet blotting system (Bio-Rad, USA) for 90 min. Membranes were kept on PBS-Tween with 5% low-fat milk O/N and incubated with 5 mM diethyl pyrocarbonate (DEPC), to inhibit binding of *S*. *aureus* protein A to IgG [53] and rabbit polyclonal anti PBP2a antibody (raised against the synthetic peptide NH2-CGSKKFEKGMKKLGVGEDIPSDYPF; RayBiotech) at 1:1000 dilution for 1 hour. After two washes the membranes were incubated with the anti-rabbit secondary antibody conjugated to horseradish peroxidase (PerkinElmer, USA) at 1:5000 dilution for 1 hour. The chemiluminiscent signal was detected using Western Lightning Plus-ECL (PerkinElmer) and CL-XPosure film (Thermo Scientific). The membrane was incubated in stripping buffer (62.5 mM Tris-HCL pH 6.7, 100 mM β -mercaptoethanol, 2% SDS) at 50°C for 30 min and re-hybridized with 5mM DEPC and polyclonal antibody raised against the amidase domain of *S*. *aureus* Atl protein, at 1:1000 dilution for 5h.

### Accession numbers

The raw reads of the 106 isolates analyzed in this study and the closed genome of *S*. *fleuretti* were deposited in ENA with the following accession number PRJEB18761.

## Supporting information

S1 Table Epidemiological information of all strains studied. The distribution of the different *mecA* homologue alleles in the population of isolates studied is also shown. Phylogenetic group (*S*. *sciuri sciuri*, *S*. *sciuri rodentius*, S, *sciuri carnaticus*, *S*. *sciuri* new subspecies group 1, *S*. *sciuri* new subspecies group 2, *S*. *vitulinus*, *S*. *fleurettii*), origin of the strains and oxacillin MIC are also shown. β-lactam resistant strains are highlighted in bold. NB: non-binding domain: TP: transpeptidase domain.
(DOCX)

S2 Table Percentage of nucleotide identity and size of the core genes of the 76 *S*. *sciuri* isolates from this study. (XLSX)

S3 Table Marginal likelihoods for the strict, fixed, random and uncorrelated molecular clock models under a constant size population model. (XLSX)

S4 Table Bayes factors for the strict, fixed, random and uncorrelated molecular clock models under a constant size population model. (XLSX)

S1 Fig Phylogenetic analysis of the *mecA* homologues nucleotide sequence. *mecA* homologues. The sequences of each *mec* homologue gene was extracted from the de novo assembly contigs and aligned with ClustalW. The tree was performed with UPGMA method, under the Jukes-Cantor substitution model, with a bootstrap of 100 replicates. The unrooted tree is shown (A). Identification of recombination events among *mecA1* alleles. The recombinant parts of *mecA1* alleles are clustered apart from the remaining portion of the allele. Moreover, a color code is applied to identity the putative major parents that were involved in the recombination events (B).
(TIF)

S2 Fig Distribution of oxacillin MICs of *S*. *sciuri* (A) and *S*. *vitulinus* (B) as determined by Etest. “Wild-type” *S*. *sciuri* strains: *S*. *sciuri* strains carrying *mecA1* only; “Resistant” *S*. *sciuri* strains: *S*. *sciuri* strains carrying *mecA1* and *mecA*. “Wild-type” *S*. *vitulinus* strains: *S*. *vitulinus* strains carrying *mecA2*; “Resistant” *S*. *vitulinus* strains: *S*. *vitulinus* strains carrying *mecA*.
(TIF)

S3 Fig Phylogenetic analysis of the core genome of isolates belonging to the *sciuri* group. Unrooted phylogenetic tree based on the number of SNP differences found among the predicted core genome of the strains. The reference genome used was *S*. *fleurettii* 402567. *S*. *fleurettii* (A). *S*. *vitulinus* (B). *S*. *sciuri* (C).
(TIF)
