# Supplementary material for: Evidence for the evolutionary steps leading to mecA-mediated β-lactam resistance in staphylococci
Source: PLoS Genet. 2017 Apr 10;13(4):e1006674. doi: 10.1371/journal.pgen.1006674 (PMC5402963; doi:10.1371/journal.pgen.1006674)
Supplement: S1 Table — The distribution of the different mecA homologue alleles in the population of isolates studied is also shown. Phylogenetic group (S. sciuri sciuri, S. sciuri rodentius, S, sciuri carnaticus, S. sciuri new subspecies group 1, S. sciuri new subspecies group 2, S. vitulinus, S. fleurettii), origin of the strains and oxacillin MIC are also shown. β-lactam resistant strains are highlighted in bold. NB: non-binding domain: TP: transpeptidase domain. (DOCX) [file pgen.1006674.s001.docx]

**S1 Table.** Epidemiological information of all strains studied. The distribution of the different *mecA* homologue alleles in the population of isolates studied is also shown. Phylogenetic group (*S. sciuri* *sciuri*, *S. sciuri* *rodentius*, S, *sciuri* *carnaticus*, *S. sciuri* new subspecies group 1, *S. sciuri* new subspecies group 2, *S. vitulinus* and *S. fleurettii*), origin of the strains and oxacillin MIC are also shown. β-lactam resistant strains are highlighted in bold. NB: non-binding domain: TP: transpeptidase domain.

| **Strain** | **Phylogenetic group** | ***mec* homologues allele** | **NB allele** | **TP allele** | **MIC (µg/ml)** | **Year of isolation** | **Origin** | **Geographic location** |
| --- | --- | --- | --- | --- | --- | --- | --- | --- |
| **JUG1** | *S. sciuri* new 1 | *mecA1* 16/*mecA* 7 | *mecA1* 11/ *mecA* 2 | *mecA1* 30/ *mecA* 2 | >256 | 2002 | Domestic dog | Czech Republic |
| **M1886** | *S. sciuri* new 1 | *mecA1* 43/*mecA* 7 | *mecA1* 18/ *mecA* 14 | *mecA1* 7/ *mecA* 2 | 64 | 2011 | Human | Denmark |
| K22 | *S. sciuri* new 1 | *mecA1* 44 | 19 | 13 | 1.5 | 1992 | Morgan horse | USA |
| **CH17** | *S. sciuri* new 2 | *mecA1* 7/*mecA* 8 | *mecA1* 14/ *mecA* 2 | *mecA1* 32/ *mecA* 3 | >256 | 2004 | Horse | Switzerland |
| **CH18** | *S. sciuri* new 2 | *mecA1* 7/*mecA* 8 | *mecA1* 14/ *mecA* 2 | *mecA1* 32/ *mecA* 3 | >256 | 2005 | Horse | Switzerland |
| **D573** | *S. sciuri* new 2 | *mecA1* 6/*mecA* 9 | *mecA1* 14/ *mecA* 4 | *mecA1* 31/ *mecA* 2 | >256 | 2007 | Human | Denmark |
| HSM805 | *S. sciuri* new 2 | *mecA1* 7 | 14 | 32 | 0.75 | 2010 | Human | Portugal |
| **HSM851** | *S. sciuri* new 2 | *mecA1* 4/*mecA* 7 | *mecA1* 12/ *mecA* 2 | *mecA1* 24/ *mecA* 2 | 16 | 2010 | Human | Portugal |
| **JUG17** | *S. sciuri* new 2 | *mecA1* 4 | 12 | 24 | >256 | 2002 | Human | Yugoslavia |
| JUG2 | *S. sciuri* new 2 | *mecA1* 15 | 10 | 29 | 0.75 | 2002 | Domestic dog | Yoguslavia |
| K132 | *S. sciuri* new 2 | *mecA1* 5 | 13 | 24 | 0.5 | 1976 | Howler monkey | Panama |
| K141 | *S. sciuri* new 2 | *mecA1* 8 | 15 | 24 | 1 | 1992 | California mouse | USA |
| K148 | *S. sciuri* new 2 | *mecA1* 3 | 14 | 25 | 0.38 | 1992 | Bottlenose dolphin | USA |
| K2 | *S. sciuri* new 2 | *mecA1* 8 | 15 | 24 | 1 | 1992 | Beef tongue | USA |
| K20 | *S. sciuri* new 2 | *mecA1* 13 | 15 | 24 | 1 | 1992 | Bottlenose dolphin | USA |
| K24 | *S. sciuri* new 2 | *mecA1* 2 | 14 | 24 | 0.75 | 1992 | Morgan horse | USA |
| K51 | *S. sciuri* new 2 | *mecA1* 8 | 15 | 24 | 1.5 | 1971 | Human | USA |
| K61 | *S. sciuri* new 2 | *mecA1* 14 | 15 | 28 | 1.5 | 1992 | Pilot whale | USA |
| K69 | *S. sciuri* new 2 | *mecA1* 9 | 16 | 24 | 1 | 1986 | Human | USA |
| KLO59 | *S. sciuri* new 2 | *mecA1* 10 | 15 | 24 | 2 | 1972 | Domestic dog | USA |
| **M1234** | *S. sciuri* new 2 | *mecA1* 6/*mecA* 9 | *mecA1* 14/ *mecA* 4 | *mecA1* 31/ *mecA* 2 | >256 | 2009 | Human | Denmark |
| **M1653** | *S. sciuri* new 2 | *mecA1* 6/*mecA* 9 | *mecA1* 14/ *mecA* 4 | *mecA1* 31/ *mecA* 2 | >256 | 2010 | Human | Denmark |
| **M2276** | *S. sciuri* new 2 | *mecA1* 6/*mecA* 9 | *mecA1* 14/ *mecA* 4 | *mecA1* 31/ *mecA* 2 | >256 | 2011 | Human | Denmark |
| **M2590** | *S. sciuri* new 2 | *mecA1* 6/*mecA* 9 | *mecA1* 14/ *mecA* 4 | *mecA1* 31/ *mecA* 2 | >256 | 2012 | Human | Denmark |
| **M2710** | *S. sciuri* new 2 | *mecA1* 4/*mecA* 7 | *mecA1* 12/ *mecA* 2 | *mecA1* 24/ *mecA* 2 | >256 | 2012 | Human | Denmark |
| **M692** | *S. sciuri* new 2 | *mecA1* 6/*mecA* 9 | *mecA1* 14/ *mecA* 4 | *mecA1* 31/ *mecA* 2 | 96 | 2007 | Human | Denmark |
| SS16 | *S. sciuri* new 2 | *mecA1* 1 | 14 | 24 | 0.75 | 1996 | Human | Portugal |
| SS18 | *S. sciuri* new 2 | *mecA1* 12 | 15 | 26 | 0.75 | 1996 | Human | Portugal |
| SS23 | *S. sciuri* new 2 | *mecA1* 11 | 15 | 27 | 1 | 1997 | Human | Portugal |
| SS24 | *S. sciuri* new 2 | *mecA1* 6 | 14 | 31 | 0.75 | 1997 | Human | Portugal |
| SS27 | *S. sciuri* new 2 | *mecA1* 12 | 15 | 26 | 0.75 | 1998 | Human | Portugal |
| SS3 | *S. sciuri* new 2 | *mecA1* 6 | 14 | 31 | 1 | 1996 | Human | Portugal |
| SS5 | *S. sciuri* new 2 | *mecA1* 8 | 15 | 24 | 1 | 1996 | Human | Portugal |
| 11/01 | *S. sciuri carnaticus* | *mecA1* 22 | 3 | 21 | 0.5 | 2002 | Human | Czech Republic |
| CCUG38359 | *S. sciuri carnaticus* | *mecA1* 23 | 2 | 21 | 0.75 | 2002 | Human | Sweden |
| K11 | *S. sciuri carnaticus* | *mecA1* 42 | 17 | 15 | 0.75 | 1990 | Veal leg | USA |
| K116 | *S. sciuri carnaticus* | *mecA1* 41 | 17 | 15 | 0.75 | 1992 | Beef lips | USA |
| K12 | *S. sciuri carnaticus* | *mecA1* 41 | 17 | 15 | 1 | 1992 | Arabian horse | USA |
| K16 | *S. sciuri carnaticus* | *mecA1* 41 | 17 | 15 | 0.75 | 1992 | Jersey calf | USA |
| K163 | *S. sciuri carnaticus* | *mecA1* 24 | 4 | 23 | 0.75 | 1992 | Holstein cow | USA |
| K30 | *S. sciuri carnaticus* | *mecA1* 25 | 5 | 22 | 1.5 | 1992 | Jersey cattle heifer | USA |
| K31 | *S. sciuri carnaticus* | *mecA1* 41 | 17 | 15 | 0.5 | 1992 | Jersey cattle calf | USA |
| K32 | *S. sciuri carnaticus* | *mecA1* 41 | 17 | 15 | 0.75 | 1992 | Jersey cattle calf | USA |
| K33 | *S. sciuri carnaticus* | *mecA1* 41 | 17 | 15 | 0.75 | 1992 | Jersey cattle heifer | USA |
| CH16 | *S. sciuri rodentius* | *mecA1* 19/*mecA* 7 | *mecA1* 7/ *mecA* 2 | *mecA1* 17/ *mecA* 2 | 24 | 2004 | Horse | Switzerland |
| K10 | *S. sciuri rodentius* | *mecA1* 20 | 6 | 19 | 0.75 | 1992 | European red squirrel | USA |
| K125 | *S. sciuri rodentius* | *mecA1* 18 | 8 | 16 | 1 | 1992 | Cotton rat | USA |
| K27 | *S. sciuri rodentius* | *mecA1* 18 | 8 | 16 | 0.75 | 1992 | Norway rat | USA |
| K29 | *S. sciuri rodentius* | *mecA1* 20 | 6 | 19 | 0.75 | 1992 | Norway rat | USA |
| **K3** | *S. sciuri rodentius* | *mecA1* 17/*mecA* 10 | *mecA1* 6/ *mecA* 3 | *mecA1* 18/ *mecA* 1 | >256 | 1992 | Neonatal ward | Mozambique |
| **K4** | *S. sciuri rodentius* | *mecA1* 17 | 6 | 18 | >256 | 1992 | Human | Mozambique |
| **K5** | *S. sciuri rodentius* | *mecA1* 17 | 6 | 18 | 25 | 1992 | Human | Mozambique |
| **K6** | *S. sciuri rodentius* | *mecA1* 42/*mecA* 7 | *mecA1* 17/ *mecA* 2 | *mecA1* 15/ *mecA* 2 | >256 | 1992 | Human | Mozambique |
| **K7** | *S. sciuri rodentius* | *mecA1* 17 | 6 | 18 | >256 | 1992 | Human | Mozambique |
| K83 | *S. sciuri rodentius* | *mecA1* 21 | 9 | 20 | 0.75 | 1992 | Human | Czech Republic |
| SS34 | *S. sciuri rodentius* | *mecA1* 20 | 6 | 19 | 0.75 | 1996 | Human | Portugal |
| **SS37** | *S. sciuri rodentius* | *mecA1* 17 | 6 | 18 | 25 | 1996 | Human | Portugal |
| **SS41** | *S. sciuri rodentius* | *mecA1* 17 | 6 | 18 | 3 | 1996 | Human | Portugal |
| K1 | *S. sciuri sciuri* | *mecA1* 36 | 25 | 7 | 2 | 1972 | Eastern grey squirrel | USA |
| K105 | *S. sciuri sciuri* | *mecA1* 34 | 20 | 12 | 1 | 1971 | Human | USA |
| K13 | *S. sciuri sciuri* | *mecA1* 27 | 20 | 5 | 0.5 | 1992 | Eastern grey squirrel | USA |
| K139 | *S. sciuri sciuri* | *mecA1* 40 | 21 | 10 | 1 | 1992 | Holstein cow | USA |
| K14 | *S. sciuri sciuri* | *mecA1* 39 | 20 | 11 | 0.75 | 1992 | Eastern harvest mouse | USA |
| K140 | *S. sciuri sciuri* | *mecA1* 28 | 20 | 4 | 1 | 1972 | Opossum | USA |
| K142 | *S. sciuri sciuri* | *mecA1* 35 | 24 | 7 | 1 | 1992 | Horse | USA |
| K143 | *S. sciuri sciuri* | *mecA1* 35 | 24 | 7 | 1 | 1972 | Racoon | USA |
| K144 | *S. sciuri sciuri* | *mecA1* 33 | 20 | 9 | 1 | 1992 | Jersey calf | USA |
| K149 | *S. sciuri sciuri* | *mecA1* 31 | 1 | 2 | 0.75 | 1972 | Eastern grey squirrel | USA |
| K21 | *S. sciuri sciuri* | *mecA1* 32 | 22 | 9 | 0.5 | 1993 | Pilot whale | USA |
| K23 | *S. sciuri sciuri* | *mecA1* 35 | 24 | 7 | 0.75 | 1992 | Red kangaroo | USA |
| K25 | *S. sciuri sciuri* | *mecA1* 29 | 23 | 3 | 0.5 | 1992 | Prairie vole | USA |
| KLO56 | *S. sciuri sciuri* | *mecA1* 38 | 20 | 8 | 0.75 | 1972 | Opossum | USA |
| KLO58 | *S. sciuri sciuri* | *mecA1* 26 | 20 | 6 | 0.75 | 1972 | Squirrel monkey | USA? |
| KLO63 | *S. sciuri sciuri* | *mecA1* 37 | 24 | 7 | 1 | 1972 | Eastern grey squirrel | USA |
| KLO64 | *S. sciuri sciuri* | *mecA1* 30 | 20 | 1 | 2 | 1972 | Southern flying squirrel | USA |
| **M1640** | *S. sciuri sciuri* | *mecA1* 37/*mecA* 7 | *mecA1* 24/ *mecA* 2 | *mecA1* 7/ *mecA* 2 | 96 | 2010 | Human | Denmark |
| H39 | *S. vitulinus* | *mecA* 4 | 5 | 4 | 0.75 | 2005 | Horse | Denmark |
| H91 | *S. vitulinus* | *mecA* 7 | 2 | 2 | 2 | 2005 | Horse | Denmark |
| 401946 | *S. vitulinus* | *mecA* 4 | 5 | 4 | 2 | 2004 | Horse | The Netherlands |
| CH1 | *S. vitulinus* | *mecA* 7 | 2 | 2 | 1 | 2005 | Horse | Switzerland |
| **CH2** | *S. vitulinus* | *mecA* 4 | 5 | 4 | 4 | 2004 | Horse | Switzerland |
| CH3 | *S. vitulinus* | *mecA* 7 | 2 | 2 | 0.75 | 2005 | Horse | Switzerland |
| CH4 | *S. vitulinus* | *mecA* 4 | 5 | 4 | 0.75 | 2004 | Horse | Switzerland |
| **CH5** | *S. vitulinus* | *mecA* 4 | 5 | 4 | >256 | 2005 | Horse | Switzerland |
| CH6 | *S. vitulinus* | *mecA* 4 | 5 | 4 | 1.5 | 2004 | Horse | Switzerland |
| CH7 | *S. vitulinus* | *mecA2* 1 | 1 | 1 | 0.75 | 2004 | Horse | Switzerland |
| CH8 | *S. vitulinus* | *mecA2* 2 | 1 | 2 | 0.19 | 2005 | Horse | Switzerland |
| CH9 | *S. vitulinus* | *mecA2* 2 | 1 | 2 | 0.5 | 2004 | Horse | Switzerland |
| CH10 | *S. vitulinus* | *mecA2* 2 | 1 | 2 | 0.5 | 2004 | Horse | Switzerland |
| CH11 | *S. vitulinus* | *mecA2* 1 | 1 | 1 | 0.5 | 2005 | Horse | Switzerland |
| CH12 | *S. vitulinus* | *mecA2* 2 | 1 | 2 | 0.5 | 2004 | Horse | Switzerland |
| CH13 | *S. vitulinus* | *mecA2* 2 | 1 | 2 | 0.1 | 2004 | Horse | Switzerland |
| CH14 | *S. vitulinus* | *mecA2* 2 | 1 | 2 | 0.5 | 2005 | Horse | Switzerland |
| **CH15** | *S. vitulinus* | *mecA2* 2 | 1 | 2 | >256 | 2004 | Horse | Switzerland |
| **402567** | *S. fleurettii* | *mecA* 5 | 6 | 4 | >256 | 2004 | Horse | The Netherlands |
| **CH19** | *S. fleurettii* | *mecA* 1 | 1 | 6 | 8 | 2009 | Horse | Switzerland |
| **CH20** | *S. fleurettii* | *mecA* 3 | 5 | 5 | 6 | 2009 | Horse | Switzerland |
| **CH21** | *S. fleurettii* | *mecA* 2 | 5 | 4 | 4 | 2010 | Horse | Switzerland |
| CH22 | *S. fleurettii* | *mecA* 2 | 5 | 4 | 1 | 2010 | Horse | Switzerland |
| **CH23** | *S. fleurettii* | *mecA* 2 | 5 | 4 | 4 | 2010 | Horse | Switzerland |
| **CH24** | *S. fleurettii* | *mecA* 2 | 5 | 4 | >256 | 2009 | Horse | Switzerland |
| **CH25** | *S. fleurettii* | *mecA* 6 | 5 | 2 | 4 | 2010 | Horse | Switzerland |
| **CH26** | *S. fleurettii* | *mecA* 2 | 5 | 4 | >256 | 2010 | Horse | Switzerland |
| **CH27** | *S. fleurettii* | *mecA* 2 | 4 | 4 | 4 | 2010 | Horse | Switzerland |
| CH28 | *S. fleurettii* | *mecA* 2 | 5 | 4 | 1 | 2010 | Horse | Switzerland |
| **CH29** | *S. fleurettii* | *mecA* 2 | 5 | 4 | 4 | 2010 | Horse | Switzerland |
