# Supplementary figures and images for: Evidence for the evolutionary steps leading to mecA-mediated β-lactam resistance in staphylococci
Source: PLoS Genet. 2017 Apr 10;13(4):e1006674. doi: 10.1371/journal.pgen.1006674 (PMC5402963; doi:10.1371/journal.pgen.1006674)

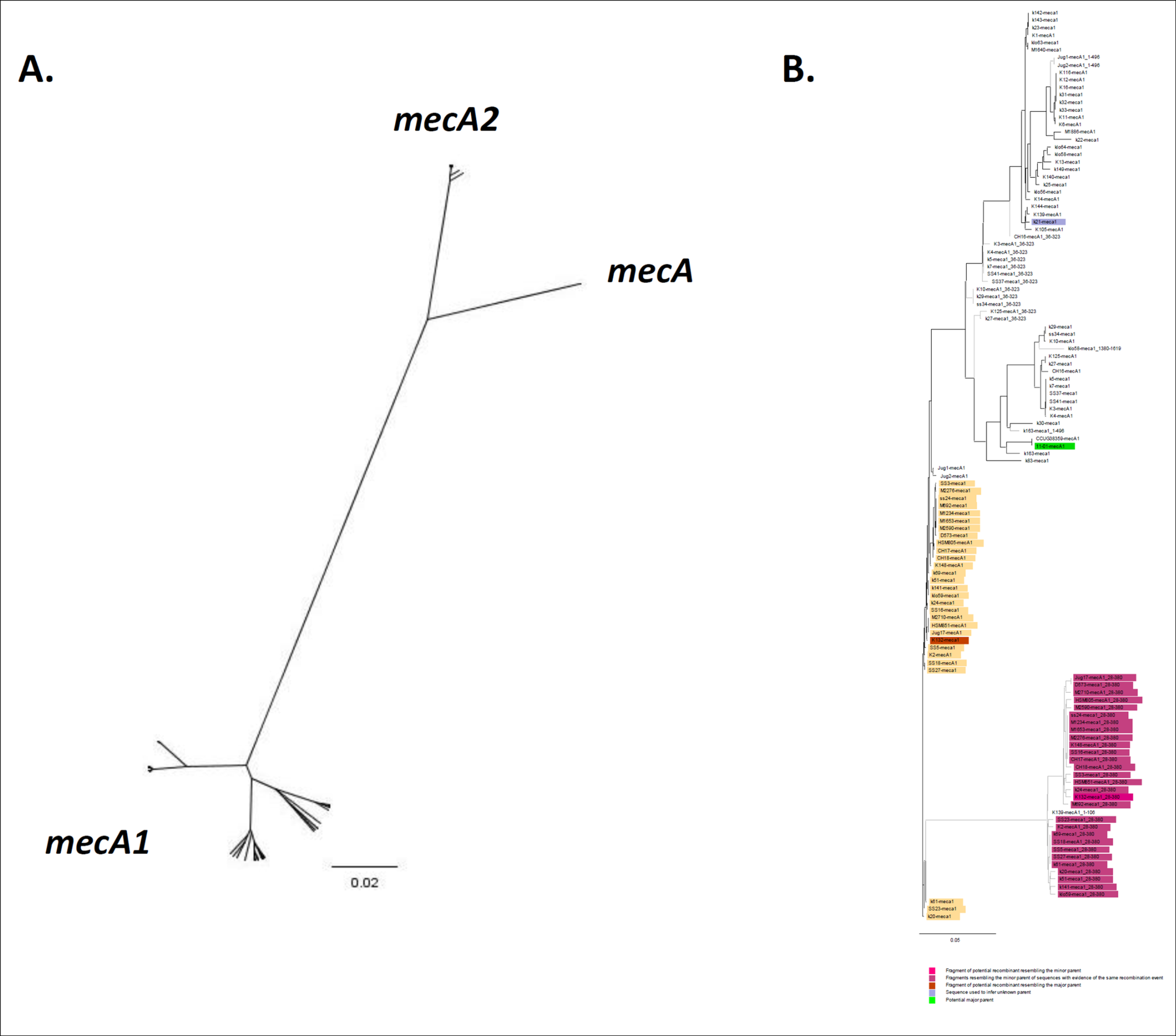

Supplement: S1 Fig — mecA homologues. The sequences of each mec homologue gene was extracted from the de novo assembly contigs and aligned with ClustalW. The tree was performed with UPGMA method, under the Jukes-Cantor substitution model, with a bootstrap of 100 replicates. The unrooted tree is shown (A). Identification of recombination events among mecA1 alleles. The recombinant parts of mecA1 alleles are clustered apart from the remaining portion of the allele. Moreover, a color code is applied to identity the putative major parents that were involved in the recombination events (B). (TIF) [file pgen.1006674.s005.tif]

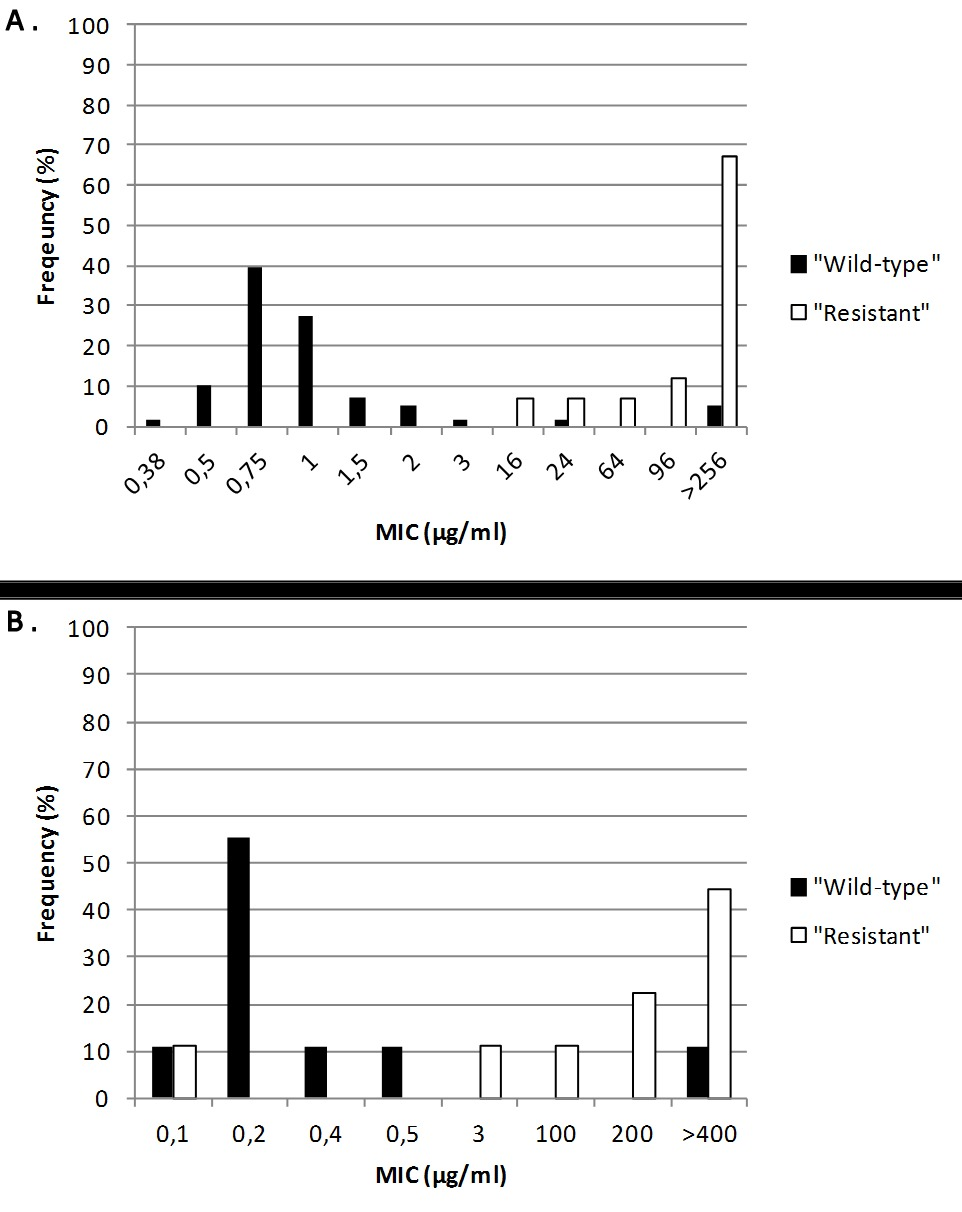

Supplement: S2 Fig — Distribution of oxacillin MICs of S. sciuri (A) and S. vitulinus (B) as determined by Etest. “Wild-type” S. sciuri strains: S. sciuri strains carrying mecA1 only; “Resistant” S. sciuri strains: S. sciuri strains carrying mecA1 and mecA. “Wild-type” S. vitulinus strains: S. vitulinus strains carrying mecA2; “Resistant” S. vitulinus strains: S. vitulinus strains carrying mecA. (TIF) [file pgen.1006674.s006.tif]

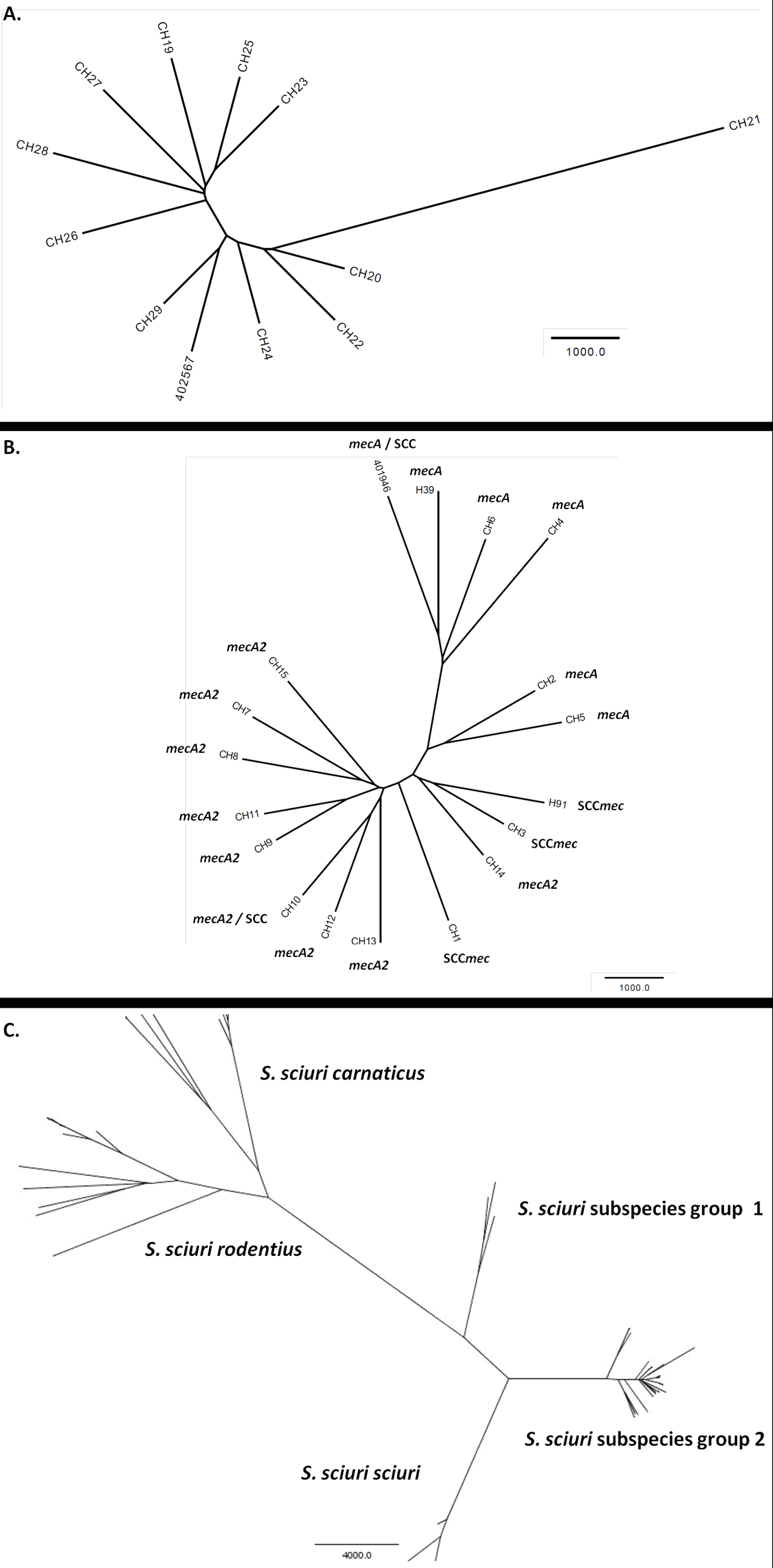

Supplement: S3 Fig — Unrooted phylogenetic tree based on the number of SNP differences found among the predicted core genome of the strains. The reference genome used was S. fleurettii 402567. S. fleurettii (A). S. vitulinus (B). S. sciuri (C). (TIF) [file pgen.1006674.s007.tif]
